# Characterization of Mice Ubiquitously Overexpressing Human 15-Lipoxygenase-1: Effect of Diabetes on Peripheral Neuropathy and Treatment with Menhaden Oil

**DOI:** 10.1155/2021/5564477

**Published:** 2021-03-15

**Authors:** Lawrence Coppey, Alexander Obrosov, Hanna Shevalye, Eric Davidson, William Paradee, Mark A. Yorek

**Affiliations:** ^1^Department of Internal Medicine, University of Iowa, Iowa City, IA 52242, USA; ^2^The Genome Editing and Viral Vector Cores, University of Iowa, Iowa City, IA 52242, USA; ^3^Department of Veteran Affairs, Iowa City Health Care System, Iowa City, IA 52246, USA; ^4^Fraternal Order of Eagles Diabetes Research Center, University of Iowa, Iowa City, IA 52242, USA

## Abstract

To rigorously explore the role of omega-3 polyunsaturated fatty acids (PUFA) in the treatment of diabetic peripheral neuropathy (DPN), we have created a transgenic mouse utilizing a Cre-lox promoter to control overexpression of human 15-lipoxygenase-1 (15-LOX-1). In this study, we sought to determine the effect of treating type 2 diabetic wild-type mice and transgenic mice ubiquitously overexpressing 15-LOX-1 with menhaden oil on endpoints related to DPN. Wild-type and transgenic mice on a C57Bl/6J background were divided into three groups. Two of each of these groups were used to create a high-fat diet/streptozotocin model for type 2 diabetes. The remaining mice were control groups. Four weeks later, one set of diabetic mice from each group was treated with menhaden oil for twelve weeks and then evaluated using DPN-related endpoints. Studies were also performed using dorsal root ganglion neurons isolated from wild-type and transgenic mice. Wild-type and transgenic diabetic mice developed DPN as determined by slowing of nerve conduction velocity, decreased sensory nerve fibers in the skin and cornea, and impairment of thermal and mechanical sensitivity of the hindpaw compared to their respective control mice. Although not significant, there was a trend for the severity of these DPN-related deficits to be less in the diabetic transgenic mice compared to the diabetic wild-type mice. Treating diabetic wild-type and transgenic mice with menhaden oil improved the DPN-related endpoints with a trend for greater improvement or protection by menhaden oil observed in the diabetic transgenic mice. Treating dorsal root ganglion neurons with docosahexanoic acid but not eicosapentaenoic acid significantly increased neurite outgrowth with greater efficacy observed with neurons isolated from transgenic mice. Targeting pathways that will increase the production of the anti-inflammatory metabolites of omega-3 PUFA may be an efficacious approach to developing an effective treatment for DPN.

## 1. Introduction

To rigorously explore the role of omega-3 polyunsaturated fatty acids (PUFA) and their metabolism as a treatment for diabetic peripheral neuropathy (DPN), we have created a transgenic mouse utilizing a Cre-lox promoter to control overexpression of human 15-lipoxygenase-1 (15-LOX-1) (Tg(CAG-eGFP,Alox15,tdTomato)#Iowa). In this study, we sought to determine the effect of feeding type 2 diabetic wild-type mice and transgenic mice ubiquitously overexpressing 15-LOX-1 a normal chow diet vs. a diet enriched in menhaden (fish) oil on endpoints related to DPN. We have also isolated dorsal root ganglion neurons from these mice to examine the effect of omega-3 PUFA on neurite growth.

Lipoxygenases can be classified into several categories based on the position of the carbon on arachidonic acid that is oxygenated [1]. Human lipoxygenases include 5-lipoxygenase, 12-lipoxygenase, and 15-lipoxygenase. Human 15-lipoxygenase was originally named arachidonate 15-lipoxygenase; however, subsequent studies identified a second human enzyme with the same activity. Therefore, the product of the human arachidonate 15-lipoxygenase gene is now called 15-LOX-1 and the product of the arachidonate 15-lipoxygenase B gene is called 15-lipoxygenase-2 [2, 3]. There is about 40% homology between these two gene products and they biologically differ in their preferred substrate [4]. It is important to specify that separate 12-lipoxygenase and 15-lipoxygenase enzymes do not exist in rodents and 12/15-lipoxygenase, the mouse homolog of 15-LOX-1, has opposing functions with 15-LOX-1 [1]. The regulation of the arachidonate 15-lipoxygenase gene is complex and nicely reviewed by Colakoglu et al. [1].

Depending on its cellular environment and available substrates at the specific time, 15-LOX-1 can be proinflammatory or anti-inflammatory. When provided with an adequate amount of omega-3 PUFA, production of inflammatory resolving mediators, resolvins, protectins, lipoxins, and maresins, is increased [5]. Our previous studies have demonstrated that treating diabetic rodents with omega-3 PUFA derived from menhaden (fish) oil slowed progression as well as reversed multiple endpoints associated with DPN [6–9]. Our studies have also shown that treating type 2 diabetic mice with resolvins also improved DPN similar to menhaden oil [10–12]. This has led us to hypothesize that anti-inflammatory mediators derived from omega-3 PUFA contribute to the beneficial effects of menhaden oil on DPN. In this study, to further explore the potential of omega-3 PUFA as a treatment of DPN, we examined the effect of menhaden oil on DPN-related endpoints in type 2 diabetic wild-type mice and mice ubiquitously overexpressing 15-LOX-1. The hypothesis being tested was that mice ubiquitously overexpressing 15-LOX-1 will be more responsive to treatment with menhaden oil and improvement in DPN.

## 2. Materials and Methods

### 2.1. Materials

Unless stated otherwise, all chemicals used in these studies were obtained from Sigma-Aldrich Chemicals Co. (St. Louis, MO).

### 2.2. Animals

**Figure 1:**
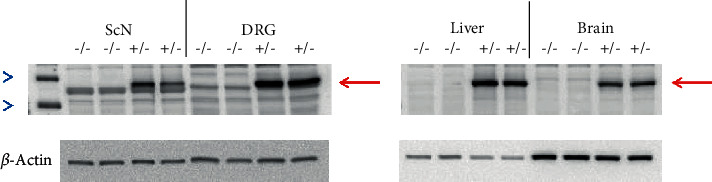
Western blot of tissues isolated from mice ubiquitously overexpressing human 15-LOX-1 (+/−) or wild-type littermates (−/−). The red arrow indicates the 15-LOX-1 protein. The molecular weight of 15-LOX-1 is 72 kDa. The blue arrowheads are markers for the 50 and 75 kDa molecular weight standards.

Wild-type C57Bl/6J mice were purchased from Jackson Laboratories. The Tg(CAG-eGFP,Alox15,tdTomato)#Iowa mouse was created in collaboration with the University of Iowa Transgenic Core Facility by virally inserting the human 15-LOX-1 gene within a Cre-lox vector. A successful founder was identified and was backcrossed for 10 generations to C57Bl/6J mice to purify the mouse genetics. To complete the ubiquitous expression of 15-LOX-1, a Tg(CAG-eGFP,Alox15,tdTomato)#Iowa male mouse was crossed with a female B6.C-Tg(CMV-cre)1Cgn/J mouse. The B6.C-Tg(CMV-cre)1Cgn/J mouse ubiquitously expresses the Cre protein which activates the Cre promoter and enables the human 15-LOX-1 gene to be fully expressed; this breeding results in one-half of the offspring expressing the gene and the 15-LOX-1 protein (+/−) while the other one-half do not express the gene (−/−). Data in Figure 1 demonstrate that +/− mice (Tg 15-LOX-1) overexpress the 15-lipoxygenase-1 protein (~72 kDa), as indicated by the red arrow, in all tissues examined including the sciatic nerve (ScN), dorsal root ganglion (DRG), liver, and brain compared to (−/−) littermate mice. Each lane was loaded with 20 *μ*g of protein of the respective tissue. The loading control for this study was *β*-actin which was equivalent for each lane as shown. Male and female mice were used in this study.

Wild-type and Tg 15-LOX-1 mice were housed in a certified animal care facility with standard diet (Harlan Teklad, #7001, Madison, WI) and water is provided ad libitum. Both male and female mice were used in these studies. Measures were taken to minimize pain or discomfort, and all experiments were conducted in accordance with international standards on animal welfare and were compliant with institutional and National Institutes of Health guidelines for use of animals (ACORP protocol 1891201). C57Bl/6J wild-type and Tg 15-LOX-1 mice at 12 weeks of age were each divided into three groups. Two of each of these groups were fed a 60 kcal% high-fat diet (D12492; Research Diets, New Brunswick, NJ). The remaining group of wild-type C57Bl/6J and Tg 15-LOX-1 mice remained on the standard diet for the duration of the study. These mice were deemed to be the control group. To create the type 2 diabetic model, the high-fat-fed mice, after 8 weeks on the high-fat diet, were treated with 100 mg/kg streptozotocin (EMD Chemicals, San Diego, CA) followed three days later with a second dose of streptozotocin (50 mg/kg). Mice with blood glucose ≥ 13.8 mM (250 mg/dl) three days later were considered to be diabetic. These groups remained on the high-fat diet for an additional four weeks. Afterwards, one group of wild-type C57Bl/6J and Tg 15-LOX-1 mice designated as the untreated diabetic group remained on the 60 kcal% high-fat diet for the entire period of the study. The other group of diabetic wild-type C57Bl/6J and Tg 15-LOX-1 mice received the 60 kcal% high-fat diet enriched with menhaden oil. This diet was produced by replacing one-half of the fat (derived primarily from lard) in the 60 kcal% high-fat diet with menhaden oil. The menhaden oil-enriched diet was prepared by Research Diets. These diets were maintained for 12 weeks, and afterwards, the following analyses were performed.

### 2.3. Behavioral Tests

Thermal sensitivity was measured using the Hargreaves method with instrumentation provided by IITC Life Science; Woodland Hills, CA (model 390G). This procedure was started by placing the mice in the confinement chamber and allowing them to acclimate to the warmed glass surface (30°C) and surroundings for 15 min. To begin the experimentation, the heat source was maneuvered so that it was under the heel of the hindpaw and the device was then activated, the process that turns on a timer and locally warms the glass surface. The experimental period was terminated when the mouse moved its paw, thereby turning off the timer and the heat source [13]. A total of five measurements were made for each mouse with the initial recording being discarded. There was a rest period of 5 min between each examination. The mean of the four measurements, reported in seconds, was used as a measure of the thermal nociceptive response latency.

Mechanical allodynia was determined by quantifying the withdrawal threshold of the hindpaw in response to stimulation with flexible von Frey filaments as previously described [14]. The data were reported in grams. The tactile response tests were repeated at least three times with a rest period of 5 minutes between tests. Both of these behavioral examinations were performed in a masked fashion on different days and completed immediately before the terminal procedures.

### 2.4. Motor and Sensory Nerve Conduction Velocity

Prior to these examinations, mice were anesthetized with Nembutal (75 mg/kg, i.p., Abbott Laboratories, North Chicago, IL). Motor and sensory nerve conduction velocities were assessed as in previous experiments [13]. The body temperature was monitored throughout the study period by using a rectal probe and regulated between 36^o^ C and 37^o^ C using a heating pad and radiant heat, which maintained an ambient temperature near the sciatic nerve [9]. Following stimulation proximally and distally, motor nerve conduction velocity was determined by using the stimulus artifact of the evoked potential, subtracting the latency measurement (in milliseconds) from the sciatic notch (proximal) from the latency measurement of the Achilles tendon (distal), and dividing the difference by the distance between the two stimulating electrodes (measured in millimeters). Sensory nerve conduction velocity was determined by measuring the distance between stimulating and recording electrodes and dividing the latency to the initial peak of negative deflection. Both motor and sensory nerve conduction velocities were reported in m/sec.

### 2.5. Corneal Innervation In Vivo

Subepithelial corneal nerves were imaged using the Rostock cornea module of the Heidelberg Retina Tomograph confocal microscope as previously described [11, 15]. Prior to experimentation, the anesthetized mouse was secured to a customized platform that allowed adjustment and positioning in three dimensions. Five random high-quality images, without overlap of the subepithelial nerve plexus of the central cornea, were acquired by finely focusing the objective lens to maximally resolve the nerve layer just under the corneal epithelium. The investigator acquiring these images was masked with respect to the identity of the mouse condition. The corneal nerve fiber length was reported as the total length of all nerve fibers and branches (in millimeters) present in the acquired images standardized for the area of the image (in square millimeters). The corneal fiber length for each animal was the mean value obtained from the acquired images and expressed as mm/mm^2^. Based on receiver operating characteristic curve analysis, the corneal nerve fiber length is the optimal parameter for diagnosing patients with diabetic neuropathy and has the lowest coefficient of variation [16].

### 2.6. Immunohistochemistry Analysis of Sensory Nerves in the Skin In Vitro

As previously described, immunoreactive nerve fiber profiles innervating the skin of the hindpaw were determined using standard confocal microscopy [13, 14]. Samples were surgically acquired and fixed and the immunostained nerve profiles were counted by two individual investigators that were masked to the sample identity. All immunoreactive profiles were normalized to the length of the epidermal specimen.

### 2.7. Analyses in Serum

In addition to 6 h fasting blood glucose, blood was also collected and serum was obtained for determination according to manufacturer's instruction of free fatty acid, triglyceride, free cholesterol, and resolvin D1 using commercial kits from Roche Diagnostics, Mannheim, Germany; Sigma-Aldrich Co., St. Louis, MO; BioVision, Mountain View, CA; and Cayman Chemical Co., Ann Arbor, MI, respectively.

### 2.8. Adult Mouse Sensory Neuron Culture

For the studies using primary neurons, dorsal root ganglia (DRG) were isolated from adult mice and neurons were grown in culture as described in [17] and modified by [10]. Neurons were isolated from 12-week old C57Bl/6J or Tg 15-LOX-1 mice that were anesthetized with Nembutal (75 mg/kg, i.p., Abbott Laboratories, North Chicago, IL) and euthanized by cervical dislocation. Following neuron isolation and initial preparation, they were suspended in complete F-12 medium (Gibco, Thermo Fisher Scientific, Grand Island, NY, USA) containing 10 mM glucose, supplemented with 10% heat-inactivated fetal bovine serum, 100 U/ml penicillin, and 100 *μ*g/ml streptomycin. No exogenous growth factors were added at this point. They were then plated on poly-D-lysine/laminin Biocoat glass coverslips (Corning, Discovery Labware Inc., Bedford, MA, USA) and were allowed to adhere for 24 hours at 37°C and 5% CO_2_. Afterwards, neurons were incubated in the experimental conditions with prewarmed complete F-12 medium with or without nerve growth factor (10 or 50 ng/ml), eicosapentaenoic acid (EPA) (40 *μ*M), docosahexaenoic acid (DHA) (40 *μ*M), resolvin D1 (50 nM), or nerve growth factor + DHA (10 ng/ml and 40 *μ*M, respectively) and incubated for 24 hours. Afterwards, the coverslips were washed twice, fixed with 4% formaldehyde (Polysciences Inc., Warrington, PA) for 20 min. Nonspecific binding was blocked by 1% bovine serum albumin, 1% normal goat serum, and 0.1% Triton X-100 in 10 mM phosphate-buffered saline at room temperature for 30 min. The blocking step was followed by incubation with neuronal class III *β*-tubulin rabbit polyclonal antibody (1 : 1,000 working dilution, Covance, Dedham, MA) overnight at 4°C and then with secondary Alexa Fluor 546-conjugated goat anti-rabbit antibody (1 : 2,000 working dilution, Invitrogen, Eugene, OR, USA) for 2 h at room temperature. Coverslips were mounted on glass slides with ProLong Gold Antifade Reagent (Life Technologies, Carlsbad, CA). Z-stack images of neuronal cells were taken in steps of 1 *μ*m for a total range of 5–6 *μ*m at 200x magnification with a Zeiss LSM710 confocal microscope and analyzed with Imaris software (version 7.6.4 ×64, Bitplane, Zurich, Switzerland). The filament tracer module of the Imaris package automatically detects cell bodies, tracks neurites in 3D, and quantifies the neurite length in *μ*m. Cell bodies were excluded from our image analyses, and the total neurite length was normalized by a number of cell bodies in each image thus producing a value of neurite length per neuron. 30–40 neurons were analyzed per condition for each mouse, and the average values were used to calculate the group means.

### 2.9. Data Analysis

Results are presented as mean ± SEM. Comparisons between groups were conducted using a one-way ANOVA and Bonferroni's test for multiple comparisons (Prism software; GraphPad, San Diego, CA). A *p* value of less than 0.05 was considered significant.

## 3. Results

Wild-type mice and mice overexpressing 15-LOX-1 weighed the same at 12 weeks of age (Table 1). At the end of the study, all mice had gained weight with diabetic mice representing a late-stage type 2 diabetic model gaining about 10–20% more weight (not significant) than their respective control mice. Treating diabetic wild-type mice with menhaden oil caused a significant increase in weight compared to control wild-type mice, but this was not observed in diabetic Tg 15-LOX-1 mice treated with menhaden oil. Fasting blood glucose levels were significantly increased in diabetic wild-type and Tg 15-LOX-1 mice independent of treatment with menhaden oil compared to their respective control mice. At the end of the study, serum-free acid levels were not changed in wild-type or Tg 15-LOX-1 mice independent of diabetes or treatment with menhaden oil. Diabetes caused a significant increase in serum triglyceride levels in diabetic wild-type and Tg 15-LOX-1 mice, and this was significantly improved by treatment with menhaden oil. Diabetes significantly increased serum cholesterol levels in diabetic wild-type mice but serum cholesterol levels were not raised significantly in diabetic Tg 15-LOX-1 mice. Treating diabetic wild-type with menhaden oil reduced serum cholesterol levels while serum cholesterol levels remained about the same in menhaden oil-treated diabetic Tg LOX-1 mice.

**Figure 2:**
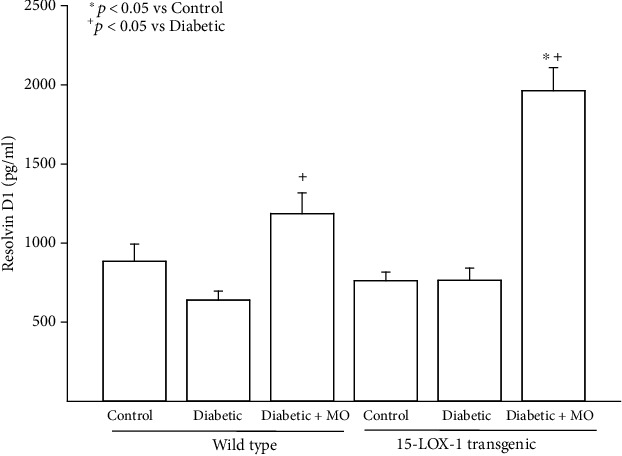
Treatment with menhaden oil of type 2 diabetic wild-type and 15-LOX-1 transgenic mice: effect on serum resolvin D1 levels. Serum resolvin D1 levels were determined as described in Section 2. Data is presented as mean ± SEM in pg/ml. The number of mice in each group was the same as presented in Table 1. ^∗^*p* < 0.05 compared to their respective control mice; ^+^*p* < 0.05 compared to their respective untreated diabetic mice.

Serum resolvin D1 levels were moderately decreased in diabetic wild-type mice compared to control wild-type mice (Figure 2). Treating diabetic wild-type mice with menhaden oil significantly increased serum resolvin D1 levels compared to diabetic wild-type mice. Serum resolvin D1 levels were about the same in control and diabetic Tg 15-LOX-1 mice. Treating diabetic Tg 15-LOX-1 mice with menhaden oil significantly increased serum resolvin D1 levels compared to control and diabetic Tg 15-LOX-1 mice.

**Figure 3:**
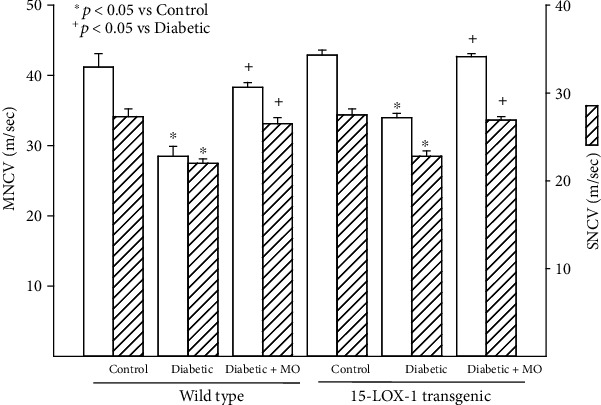
Treatment with menhaden oil of type 2 diabetic wild-type and 15-LOX-1 transgenic mice: effect on motor and sensory nerve conduction velocities. Motor and sensory nerve conduction velocities were examined as described in Section 2. The number of mice in each group was the same as described in Table 1. Data are presented as the mean ± SEM in m/sec. ^∗^*p* < 0.05 compared to their respective control mice; ^+^*p* < 0.05 compared to their respective untreated diabetic mice.

In wild-type mice as previously reported, diabetes causes a significant decrease in motor and sensory nerve conduction velocities compared to control mice, which is improved significantly when diabetic mice are treated with menhaden oil (Figure 3) [10, 12]. The pattern was similar in Tg 15-LOX-1 mice. Diabetes caused a significant decrease in both motor and sensory nerve conduction velocities that was significantly improved following treatment with menhaden oil. Comparing the data on nerve conduction velocities between wild-type and Tg 15-LOX-1 mice suggests that the decrease in motor nerve conduction velocity in diabetic mice trended to be less in Tg-15-LOX-1 mice than wild-type mice.

**Figure 4:**
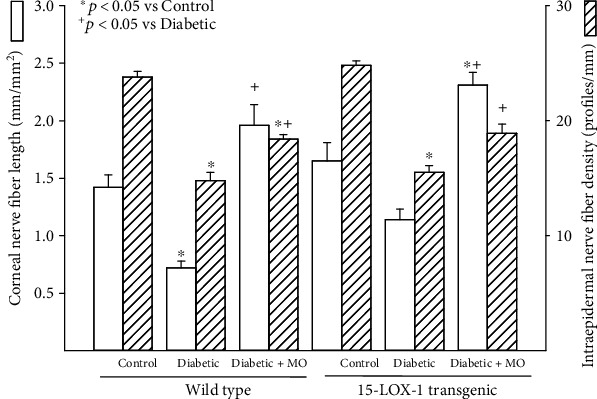
Treatment with menhaden oil of type 2 diabetic wild-type and 15-LOX-1 transgenic mice: effect on corneal nerve fiber length and intraepidermal density. Corneal nerve fiber length and intraepidermal nerve fiber density were examined as described in Section 2. The number of mice in each group was the same as described in Table 1. Data are presented as the mean ± SEM in mm/mm^2^ and profiles/mm for corneal nerve fiber length and intraepidermal nerve fiber density, respectively. ^∗^*p* < 0.05 compared to their respective control mice; ^+^*p* < 0.05 compared to respective untreated diabetic mice.

Data in Figure 4 demonstrate that in wild-type mice, diabetes causes a significant decrease in corneal nerve fiber length and intraepidermal nerve fiber density compared to control wild-type mice. Treating diabetic wild-type mice with menhaden oil caused a significant improvement in the corneal nerve fiber length and to a lesser extent intraepidermal nerve fiber density compared to untreated diabetic wild-type mice. However, intraepidermal nerve fiber density remained significantly decreased in diabetic wild-type mice treated with menhaden oil compared to control wild-type mice. When preforming these evaluations in Tg 15-LOX-1 mice, noticeable differences were observed. First, the levels of expression of sensory nerve fibers in the skin and cornea are similar for wild-type and Tg 15-LOX-1 mice. Induction of diabetes in Tg 15-LOX-1 mice caused a significant decrease in intraepidermal nerve fibers compared to that in control Tg 15-LOX-1 mice but the decrease in corneal nerve fiber length in diabetic Tg 15-LOX-1 mice was not significant compared to that in control Tg 15-LOX-1 mice. Treating diabetic Tg 15-LOX-1 mice with menhaden oil significantly improved intraepidermal nerve fiber density compared to untreated diabetic Tg 15-LOX-1 mice. Treating diabetic Tg 15-LOX-1 mice with menhaden oil also significantly increased the corneal fiber length compared to untreated diabetic Tg 15-LOX-1 mice as well as compared to control Tg 15-LOX-1 mice.

**Figure 5:**
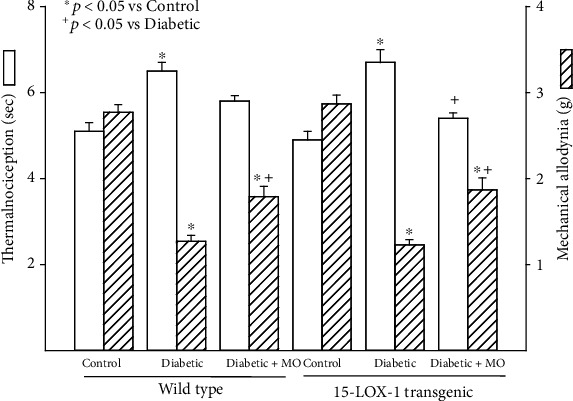
Treatment with menhaden oil of type 2 diabetic wild-type and 15-LOX-1 transgenic mice: effect on thermal nociception and mechanical allodynia. Thermal nociception and mechanical allodynia were examined as described in Section 2. The number of mice in each group was the same as described in Table 1. Data are presented as the mean ± SEM in sec and g for thermal nociception and mechanical allodynia, respectively. ^∗^*p* < 0.05 compared to their respective control mice; ^+^*p* < 0.05 compared to their respective untreated diabetic mice.

Treating wild-type diabetic mice with menhaden oil improved latency to a thermal stimulus Figure 5). Mechanical allodynia was significantly improved in diabetic wild-type mice compared to untreated diabetic wild-type mice, but the difference remained significantly impaired compared to control wild-type mice. Treating Tg 15-LOX-1 diabetic mice with menhaden oil significantly improved both latency to a thermal stimulus and mechanical allodynia compared to untreated diabetic Tg 15-LOX-1 mice. However, mechanical allodynia in menhaden oil-treated diabetic Tg 15-LOX-1 mice remained significantly impaired compared to control Tg 15-LOX-1 mice.

**Figure 6:**
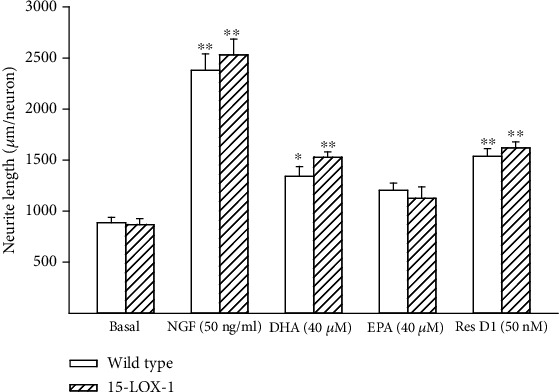
Effects of nerve growth factor (NGF), docosahexaenoic acid (DHA), eicosapentaenoic acid (EPA), or resolvin D1 (50 nM) on neurite growth by dorsal root ganglion neurons derived from wild-type and 15-LOX-1 transgenic mice. Dorsal root ganglion neurons were collected and cultured as described in Section 2. Afterwards, the effects of nerve growth factor (50 ng/ml), docosahexaenoic acid (40 *μ*M), eicosapentaenoic acid (40 *μ*M), or resolvin D1 (50 nM) on neurite outgrowth were determined. Data are presented as the mean ± SEM in *μ*m per neuron. The number of mice used per group ranged from 10 to 12. ^∗^*p* < 0.05 compared to their respective basal; ^∗∗^*p* < 0.01 compared to their respective basal.

**Figure 7:**
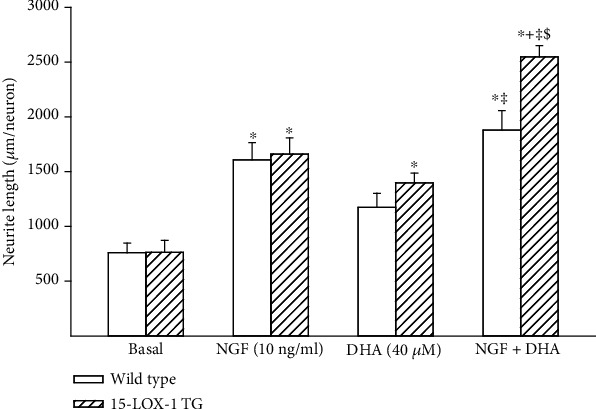
Effects of nerve growth factor (NGF), docosahexaenoic acid (DHA), or NGF + DHA on neurite growth by dorsal root ganglion neurons derived from wild-type and 15-LOX-1 transgenic mice. Dorsal root ganglion neurons were collected and cultured as described in Section 2. Afterwards, the effects of nerve growth factor (10 ng/ml), docosahexaenoic acid (40 *μ*M), or the combination of NGF + DHA on neurite outgrowth were determined. Data are presented as the mean ± SEM in *μ*m per neuron. The number of mice used per group was 9. ^∗^*p* < 0.05 compared to their respective basal; ^+^*p* < 0.05 compared to their respective NGF-treated neuron; ^‡^compared to their respective DHA-treated neurons; ^$^compared to wild-type combined NGF + DHA-treated neurons.

It has been previously reported that omega-3 PUFA, resolvin D1, and neuroprotectin D1 can increase neurite outgrowth by either dorsal root or trigeminal ganglia neurons [10, 18, 19]. In this study, we examined the effect of nerve growth factor, omega-3 PUFAs docosahexaenoic and eicosapentaenoic acids, and resolvin D1 on neurite growth by cultured primary neurons isolated from dorsal root ganglia derived from wild-type or Tg 15-LOX-1 mice. Data in Figure 1 has demonstrated that dorsal root ganglia from Tg 15-LOX-1 mice overexpress 15-lipoxygenase-1. Data in Figure 6 show that basal growth of neurites is similar for neurons isolated from wild-type or Tg 15-LOX-1 mice. Nerve growth factor treatment significantly stimulates neurite growth similarly in neurons isolated from wild-type or Tg 15-LOX-1 mice. Treating neurons with docosahexaenoic acid also significantly stimulates neurite growth with a higher degree of significance observed in neurons isolated from Tg 15-LOX-1 mice. Resolvin D1 treatment stimulated neurite growth similarly in neurons isolated from wild-type or Tg 15-LOX-1 mice. In contrast, treating neurons with eicosapentaenoic acid did not significantly increase neurite growth. In the next experiment, we examined the effect of combining a suboptimal dose of nerve growth factor with docosahexaenoic acid on neurite growth by neurons isolated from wild-type and Tg 15-LOX-1 mice (Figure 7). Nerve growth factor (10 ng/ml) significantly increased neurite growth similarly in neurons from wild-type or Tg 15-LOX-1 mice but to a lesser extent than when the dose was 50 ng/ml (Figure 6). In this study, docosahexaenoic acid significantly increased neurite growth by neurons derived from Tg 15-LOX-1 mice but not wild-type mice. When nerve growth factor and docosahexaenoic acid were combined, the effect on neurite growth by neurons isolated from wild-type mice appeared to be additive. However, the effect of the combination of nerve growth factor and docosahexaenoic acid on neurite growth by neurons derived from Tg 15-LOX-1 mice was greater than those observed with neurons from wild-type mice and those treated with combination and more than additive.

## 4. Discussion

A primary purpose of this article was to introduce the Tg human 15-lipoxygenase-1 mouse model created through the University of Iowa. When breeding these mice, we have not noticed any reproduction, growth, or developmental changes in those mice ubiquitously overexpressing human 15-LOX-1. As proof of concept, we have also performed studies demonstrating that 15-LOX-1 can be overexpressed in a tissue-specific manner (liver and sensory neurons) using CRE tissue-specific mice (data not shown). 15-Lipoxygenase-1 serves an important role in the metabolism of PUFA. Depending on the lipid source, 15-lipoxygenase-1 can generate mediators that have pro- or anti-inflammatory properties [1]. This makes 15-LOX-1 expression and regulation an attractive target for treating diseases that are impacted through inflammatory/anti-inflammatory mechanisms.

15-Lipoxygenase-1 catalyzes an integral reaction in the enzymatic pathways for the formation of both E and D series resolvins, anti-inflammatory metabolites of eicosapentaenoic acid and docosahexaenoic acid, respectively [20]. Docosahexaenoic acid can also be converted into neuroprotectin D1 through lipoxygenation catalyzed by 15-LOX-1 followed by epoxidation and hydrolysis reactions [20, 21]. Neuroprotectin D1 is another potent anti-inflammatory mediator and has been reported to be neural cell protective and has prosurvival repair signaling properties including the induction of antiapoptotic proteins and inhibition of proapoptotic proteins [20, 22]. It has been shown to have nerve regenerative properties through an experiment demonstrating regeneration of corneal nerves after topical treatment with neuroprotectin D1 following lamellar keratectomy [23]. Interestingly, in this study, the authors demonstrated similar nerve regeneration following topical treatment with pigment epithelial-derived factor and docosahexaenoic acid suggesting in vivo formation of neuroprotectin D1 by the epithelium of the cornea [18, 23]. This is consistent with reports of docosahexaenoic acid and neuroprotectin D1 displaying neuroprotective properties in the retina, brain, and central nervous system, as well as promoting neurite growth in hippocampal neurons and rat cortical neurons [19, 24–26]. Activation of neuroprotectin D1 biosynthesis following administration of docosahexaenoic acid has also been shown to attenuate cerebral ischemic injury after experimental stroke [27, 28].

In this study, we sought to determine whether ubiquitous overexpression of human 15-lipoxygenase-1 would provide greater efficacy toward peripheral nerve damage caused by type 2 diabetes after treatment with menhaden oil, a natural source of the omega-3 PUFA eicosapentaenoic acid and docosahexaenoic acid. It was hypothesized that overexpression of 15-lipoxygenase-1 would lead to a greater production of resolvins and neuroprotectin D1 and thus greater protection and regeneration of peripheral nerves following induction of hyperglycemia. The rationale for this hypothesis was derived from our previous studies that demonstrated that menhaden oil and daily administration of resolvins E or D are an effective treatment for diabetic peripheral neuropathy [6–12]. We found that treating diabetic mice ubiquitously overexpressing 15-LOX-1 with menhaden oil led to an increased production of resolvin D1 significantly greater than that observed with wild-type diabetic mice treated with menhaden oil. Treating both wild-type and Tg-15-LOX-1 mice with menhaden oil lowered blood glucose and serum triglyceride levels. These data in part agree with a recent meta-analysis of the effect of fish oil supplementation of patients with type 2 diabetes which has shown that fish oil supplementation leads to a favorable blood lipid profile but improvement in glucose control is controversial [29]. There have been reports of fish oil both improving and having no effect on insulin sensitivity. In diabetic Tg 15-LOX-1 mice treated with menhaden oil, there was an increased generation of corneal nerves and to lesser extent intraepidermal nerve fibers. We did not observe an increased sensitivity of intraepidermal nerve fiber sensation to a thermal or mechanical stimulus. This could be due to the minimal improvement in intraepidermal nerve fiber density observed when diabetic Tg-15-LOX-1 mice were treated with menhaden oil. We did not measure corneal nerve sensitivity in these mice, which is much more technically difficult due to the size of the eye than it is for rats. The observation that corneal nerve regeneration is more sensitive to menhaden oil treatment that intraepidermal nerve fibers is consistent with our previous study done in type 2 diabetic rats [6]. It is also consistent with reports from Tavakoli and colleagues that have demonstrated regeneration of corneal nerves following pancreas/renal transplant and have promoted this as an early marker of peripheral nerve recovery [30]. We also did not see any significantly greater improvement in motor or sensory nerve conduction velocity in diabetic Tg 15-LOX-1 mice treated with menhaden oil compared to diabetic wild-type mice treated with menhaden oil. This may be due to a noted limitation of the study design. In this study, we only used one concentration of menhaden oil in the diet. We have previously demonstrated that the recovery of peripheral nerve function and structure following treatment with menhaden oil is dependent on the amount of menhaden oil substituted in the diet [31]. In this study, we used the most efficacious dosing of menhaden oil [10]. Future study will determine whether treating diabetic Tg 15-LOX-1 mice with a lower concentration of menhaden oil in the diet will be more effective than the same treatment of diabetic wild-type mice toward restoring peripheral nerve function and structure due to a greater production of resolvins and neuroprotectin D1 by diabetic Tg 15-LOX-1 mice that will not occur in diabetic wild-type mice treated with a lower concentration of menhaden oil. Another limitation in the study design was that we did not establish a baseline for neuropathology for wild-type and Tg 15-LOX-1 mice at the beginning of menhaden oil treatment. This was only determined at the end of the study. Future studies with these Tg 15-LOX-1 mice will also aid in determining the mechanism(s) responsible for many of the beneficial effects attributed to omega-3 PUFA. It is widely accepted that omega-3 PUFA reduces inflammatory and oxidative stress in a wide range of diseases including cardiovascular diseases, neurodegenerative diseases, and metabolic syndrome [32]. In the heart, resolvin D1 has been reported to reduce the infarct size through a phosphoinositide 3-kinase (PI3K)/protein kinase B (Akt) mechanism [33]. There is a lot more work that needs to be done to definitively determine the mechanism of action of omega-3 PUFA, but with the ability to overexpress 15-LOX-1 in a tissue-specific manner, site-specific studies can be performed to determine the importance of this enzyme and the generation of omega-3 PUFA metabolites in vivo.

In this study, we also explored the effect of nerve growth factor, eicosapentaenoic acid, docosahexaenoic acid, resolvin D1, or the combination of nerve growth factor and docosahexaenoic acid on neurite growth by primary neurons derived from the dorsal root of wild-type and Tg 15-LOX-1 mice. These studies demonstrated using optimal doses of docosahexaenoic acid that neurite growth was greater by dorsal root ganglion neurons derived from 15-LOX-1 mice compared to wild-type mice. Furthermore, the combination of nerve growth factor and docosahexaenoic acid significantly increased neurite growth by neurons isolated from Tg 15-LOX-1 mice compared to wild-type mice. These results imply that neurons overexpressing 15-LOX-1 are primed to increase nerve regeneration when provided adequate levels of docosahexaenoic acid.

## 5. Conclusions

Clearly, the metabolism of lipids plays a critical role in health and disease. Docosahexaenoic acid treatment has been widely demonstrated to have beneficial effects in patients with coronary artery disease, asthma, rheumatoid arthritis, osteoporosis, sepsis, cancer, dry eye disease, and age-related macular degeneration [27, 34]. With regard to 15-LOX-1, its dual role as both a pathway for generation of proinflammatory mediators and anti-inflammatory mediator makes it an attractive choice for investigation into its regulation and manipulation as a target to modulate and create a healthy homeostasis. Increased production of resolvins and neuroprotectin D1 derived from omega-3 PUFA may also be an effective treatment for neuropathies of the peripheral nervous system, and discovery of approaches that can increase their production may provide new treatment options for a variety of diseases [6–12]. For example, we have shown that the combination of menhaden oil and salsalate increases the production of resolvin D1 and is a more efficacious treatment for diabetic peripheral neuropathy than menhaden oil alone [11, 31]. It has also been demonstrated that exercise promotes an epinephrine-mediated increase in 15-lipoxygenase-1 expression and resolvin D1 synthesis [35]. Continued studies along these lines are important to further advance the lipidomics of omega-3 PUFA as a treatment option for different disease conditions associated with inflammation including diabetic peripheral neuropathy.

## Figures and Tables

**Table 1:** Effect of menhaden oil treatment of diabetic wild-type (Wt) or 15-LOX-1 transgenic (Tg) mice on serum free fatty acid, triglyceride, and cholesterol levels.

Determination	Control (Wt) (14)	Control (Tg) (11)	Diabetic (Wt) (12)	Diabetic (Tg) (14)	Diabetic + MO (Wt) (14)	Diabetic + MO (Tg) (14)
Start weight (g)	20.6 ± 0.8	20.8 ± 0.5	21.0 ± 0.9	20.9 ± 0.6	20.3 ± 0.4	20.8 ± 0.8
Final weight (g)	27.8 ± 1.1	27.5 ± 1.0	34.1 ± 2.4	31.8 ± 1.3	39.6 ± 3.3^a^	33.7 ± 2.8
Fasting blood glucose (mg/dl)	195 ± 8	200 ± 10	449 ± 28^a^	463 ± 32^a^	352 ± 25^a^	401 ± 24^a^
Serum-free fatty acids (mmol/l)	0.20 ± 0.03	0.18 ± 0.02	0.17 ± 0.02	0.19 ± 0.05	0.17 ± 0.01	0.13 ± 0.01
Serum triglycerides (mg/dl)	6.2 ± 0.7	4.0 ± 0.4	8.1 ± 0.5^a^	8.4 ± 0.6^a^	4.7 ± 0.7^b^	5.0 ± 1.0^b^
Cholesterol (mg/ml)	2.6 ± 0.5	1.9 ± 0.4	5.8 ± 0.6^a^	4.2 ± 0.6	4.1 ± 0.9	4.2 ± 0.7

Data are presented as the mean ± SEM. ^a^*p* < 0.05 compared to their respective control; ^b^*p* < 0.05 compared to their respective diabetic. Number of experimental animals ().

## Data Availability

Since this work was done in part through a support of a grant from the Veterans Affairs the original data is only available upon request, interested parties can gain access to the data supporting conclusions of this study by contacting the senior author. Please email Dr. Mark Yorek at mark-yorek@uiowa.edu or mark.yorek@va.gov. The Tg human 15-lipoxygenase1 introduced in this article is available to other laboratories. Contact one of the 2 emails above for additional information.
